# Shear-Transformation Zone Activation during Loading and Unloading in Nanoindentation of Metallic Glasses

**DOI:** 10.3390/ma12091477

**Published:** 2019-05-07

**Authors:** Karina E. Avila, Stefan Küchemann, Iyad Alabd Alhafez, Herbert M. Urbassek

**Affiliations:** 1 Physics Department, University Kaiserslautern, Erwin-Schrödinger-Straße, D-67663 Kaiserslautern, Germany; kavila@rhrk.uni-kl.de (K.E.A.); s.kuechemann@physik.uni-kl.de (S.K.); alhafez@rhrk.uni-kl.de (I.A.A.); 2 Research Center OPTIMAS, University Kaiserslautern, Erwin-Schrödinger-Straße, D-67663 Kaiserslautern, Germany

**Keywords:** molecular dynamics, nanoindentation, metallic glass

## Abstract

Using molecular dynamics simulation, we study nanoindentation in large samples of Cu–Zr glass at various temperatures between zero and the glass transition temperature. We find that besides the elastic modulus, the yielding point also strongly (by around 50%) decreases with increasing temperature; this behavior is in qualitative agreement with predictions of the cooperative shear model. Shear-transformation zones (STZs) show up in increasing sizes at low temperatures, leading to shear-band activity. Cluster analysis of the STZs exhibits a power-law behavior in the statistics of STZ sizes. We find strong plastic activity also during the unloading phase; it shows up both in the deactivation of previous plastic zones and the appearance of new zones, leading to the observation of pop-outs. The statistics of STZs occurring during unloading show that they operate in a similar nature as the STZs found during loading. For both cases, loading and unloading, we find the statistics of STZs to be related to directed percolation. Material hardness shows a weak strain-rate dependence, confirming previously reported experimental findings; the number of pop-ins is reduced at slower indentation rate. Analysis of the dependence of our simulation results on the quench rate applied during preparation of the glass shows only a minor effect on the properties of STZs.

## 1. Introduction

Plastic strain of metallic glasses well below the glass transition temperature is known to be localized into shear bands [1,2,3,4]. The core of such shear bands extends over 10–200 nm [5]. However, in the vicinity of shear bands, the deformation extends up to three orders of magnitude further [6,7,8].

After accommodating a certain plastic strain, shear bands have the tendency to form cracks eventually leading to failure and thus, particularly at temperatures well below the glass transition, limiting the applications of metallic glasses as structural materials. The origin of shear-band formation is the correlated activation of local plastic shear events, often called shear transformation zones (STZs), involving hundreds of atoms [9,10,11]. Once STZs overcome a percolation threshold, they interact and align in a two-dimensional plane [10,12]. The understanding of the role and interaction of STZs is of paramount importance for fundamental concepts and for the control of yielding, ductility and failure.

The extension of the deformation around a shear band can be probed, for instance, via nanoindentation [7] since it is sensitive to small variations in the elastic and plastic properties [13]. During nanoindentation, the activation of STZs manifests itself in the load-displacement curve as a sudden displacement increase in load-controlled measurements, or as a sudden load drop in displacement-controlled measurements, which are called pop-ins. The occurrence of the first pop-in is indicative of the onset of plasticity which contains information about fracture toughness [14,15,16,17,18]. The pop-ins are seen as serrated activity in the load-depth curve and have been studied in the perspective of a stick-slip mechanism or avalanche dynamics [19,20]. Also for other materials, avalanche statistics have been important for understanding how local regions communicate with each other under different stimuli [21,22,23]. Most recently, the transition from STZs to shear bands has been studied in the context of directed percolation [12,24]. During unloading, on the other hand, experiments usually report a smooth decrease in displacement [16,18].

In this work, we perform molecular dynamics (MD) simulations of nanoindentation in a Cu${}_{64.5}$Zr${}_{35.5}$ glass in order to study plastic deformation during loading and unloading. On an atomistic level, we find groups of atoms which cooperatively perform drastic plastic deformation during loading, but despite their large deformation they return to their initial configuration during unloading. On the other hand, as a macroscopic response, our results qualitatively agree with the so-called cooperative-shear model [11]. We measure the STZ sizes at maximum indentation depth using a cluster analysis. We find that the complementary cumulative distribution of STZ sizes follows a power-law distribution with a temperature dependent cutoff of the distribution tails. At room temperature and below, we also observe activation from STZs to shear-band formation. In addition we demonstrate that during the unloading process the system undergoes a significant amount of heterogeneous strain, similar to the loading process, which itself leads to the activation of new STZs in previously undistorted parts of the sample. We find that the size distribution of STZs during unloading is very temperature dependent. Also, the statistics of the STZs during unloading follow the same power law found during loading. These findings add new aspects in the understanding of deformations of metallic glasses and highlight the influence of the unloading process on the post-test deformation pattern.

## 2. Simulation Details

We used the open-source code LAMMPS [25] to simulate the binary-composition metallic glass Cu${}_{64.5}$Zr${}_{35.5}$. The sample consisted of *N* = 5,619,712 atoms contained in a cubic simulation box of edge length *L*. Its size varied from L=450.01 Å for the lowest temperature to 457.46 Å, for the highest temperature. In turn, the density of the quenched glass, $\rho =N/L^{3}$, took the values of 0.0616 to 0.0599, in agreement with previous studies [26], and the potential energy per atom from −4.43 eV/atom to −4.28 eV/atom. The atomic interactions were modeled by the embedded atom method (EAM) potential presented in [27]. A crystalline mixture was first heated to a temperature T=2000 K for a time period of 500 ps and then cooled to the final temperature with a quenching rate of 0.01 K/ps, to obtain the metallic glass. A slow quenching rate was preferable for the purpose of studying plastic deformation [26,28]. We therefore dedicate Appendix A to explore how our results are affected by the quenching rate. The glass transition temperature $T_{g}$ for this particular composition and potential was in the range of 800 K to 1000 K [29]. Here, we simulated samples at eight different temperatures. We included T=1000 K, which was above the glass transition temperature, to observe the nanoindentation effects in the supercooled-liquid regime. During the preparation of the sample, periodic boundary conditions were applied in all directions and an isobaric ensemble (at vanishing pressure) with a Nose-Hoover thermostat was used. Once the final temperature was reached, the system was left to relax for a total time of 200 ps with periodic boundary conditions.

After sample preparation, we applied periodic boundary conditions in the lateral directions and free boundary conditions on the top surface and let the system relax for an additional time of 300 ps before indentation. After relaxation, the stress components in all Cartesian directions are below 50 MPa at all temperatures. We fixed 10 atomic layers at the bottom of the sample in order to mimic the immobile bulk of a metallic glass in a real experiment and to avoid center-of-mass translation of the entire sample. We used a spherical indenter of radius R=10 nm. The purely repulsive force exerted by the indenter on the system is given by

(1) $$F\left(r\right)=K{\left(r-R\right)}^{2},$$

where *r* is the distance of an atom to the center of the indenter [30]. The use of such ‘external force fields’ to mimic the action of a rigid indenter on a substrate is standard in molecular dynamics simulations of nanoindentation [31]; the use of an atomistic indenter would only be appropriate if effects of indenter wear or chemical interactions between indenter and substrate were to be modeled; this was not the case here. The stiffness constant of the indenter has been set to K=10 eV/Å${}^{3}$. During indentation we kept the temperature fixed by using an NVT ensemble. The open-source visualization tool OVITO [32] was used in our work to observe the atomistic configurations and to perform a cluster analysis of STZs.

## 3. Results

### 3.1. Hardness and Pit Shape

**Figure 1:**
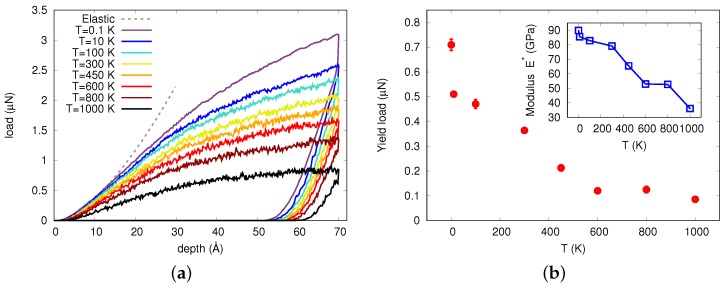
(**a**) Indentation load vs. indentation depth for different temperatures *T*. The dashed gray curve corresponds to the fitted elastic response, Equation (2). (**b**) Value of the load at the yielding point as a function of temperature. Inset: reduced elastic modulus $E^{*}$ as a function of temperature.

Figure 1a shows the load against indentation depth for different temperatures below and at the glass transition temperature at an indentation rate of 20 m/s, measured in an indentation-depth-controlled mode. The dashed gray curve in this figure corresponds to the Hertzian elastic response fitted to the T=0.1 K curve, [33,34]

(2) $$F=\frac{4}{3}R^{1/2}E^{*}d^{3/2}.$$

Here *F* is the load, *d* the indentation depth, *R* is the indenter radius and $E^{*}=E/\left(1-\nu^{2}\right)$ is the reduced elastic modulus, where *E* is the Young modulus and ν the Poisson ratio of the glass. From a fit of our data (at room temperature) we obtain $E^{*}=79.1$ GPa; this value compares favorably with experimental data which provided E=92 GPa and ν=0.352 at room temperature [35], hence $E^{*}=105$ GPa.

Figure 1a features frequent pop-ins, or load drops, which occur at all studied temperatures. This effect has been also observed in nanoindentation experiments performed in other metallic glasses [14,15]. This figure also shows that the yielding point—i.e., the deviation from the elastic curve, where the first pop-in appears—occurs later for lower temperatures. To estimate the yielding point we subtracted the fitted Hertzian elastic behavior, Equation (2), from our data, and considered the yielding point to occur when the difference was greater than 0.04μN, i.e., when the difference surpassed the fluctuations in the elastic regime. The estimated values of load at the yielding point are shown in Figure 1b. Note the strong change of yield load between 0.1 and 10 K; such a behavior was quite different from that of crystalline materials which show only small changes—in the range of 10%—between 0 K and room temperature [31]. The inset of this figure shows the fitted values of the reduced elastic modulus $E^{*}$ at the yielding point as a function of temperature. They demonstrate a strong decrease of the glass stiffness with increasing temperature; towards the glass transition temperature, the stiffness decreased by around 50%.

Moreover, it is interesting to note that there was also a change in the slope of the load-depth curve during unloading, which was most noticeable at the end of the unloading curve at temperatures of 10 K and of 100 K. In accordance to the displacement jumps during loading, these features are called pop-outs. In crystals such as Si, pop-outs may be related to pressure-induced phase transformations [36]. To our knowledge, in metallic glasses, this feature has not been reported in experiments and it has not been explored in simulations. The structural origin of the pop-outs is further discussed in relation to the non-affine squared displacement (NASD) below.

**Figure 2:**
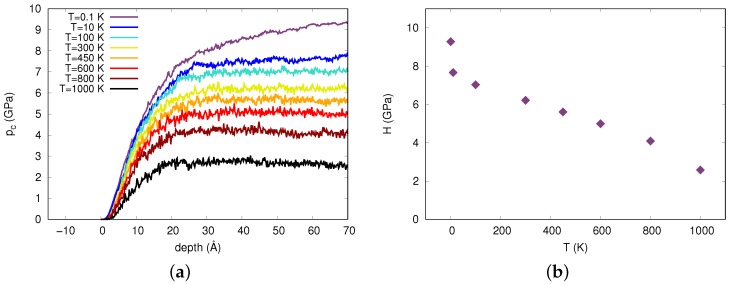
(**a**) Contact pressure as a function of indentation depth obtained for different temperatures. (**b**) Hardness as a function of temperature averaged from the plateau of panel (**a**).

**Figure 3:**
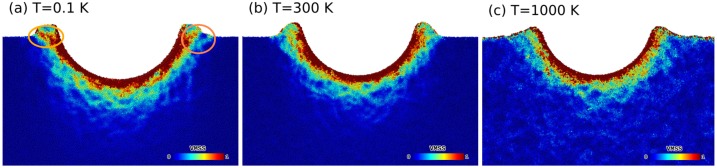
Cross-sectional view of the von–Mises shear strain (VMSS) at maximum indentation depth (70 Å) for temperatures of (**a**) T=0.1 K, (**b**) T=300 K and (**c**) T=1000 K. The orange circles in (**a**) highlight wing-like shear bands.

From the load-displacement curve, the contact pressure can be determined by the relation

(3) $$p_{c}=F/A_{c},$$

where $A_{c}$ is the contact area which was determined using the elliptic-area method described in [37]. This method calculates the area of an ellipse by using the minimum and maximum position of the atoms, both in *x* and *y* directions, contained within a shell separated by a distance $r_{c}$ from the indenter. For our analysis we used $r_{c}=7.6$ Å. However, we excluded pile-up atoms, i.e., those above the original surface, to contribute to the area determination. The material hardness *H* was the asymptotic value of the contact pressure obtained at large indention depths. Figure 2a shows the dependence of $p_{c}$ on indentation depth as determined from Equation (3). At all temperatures below $T_{g}$ the contact pressure increased as a function of indentation depth and then saturated after a certain value; only at the lowest temperature, 0.1 K, the contact pressure keeps increasing with indentation depth. We attributed this behavior to the activation of shear bands, which are particularly pronounced at the lowest temperature, see Figure 3 and its discussion below; these shear bands produced hardening in their vicinity. Further evidence for this argument was provided by the fact that a glass material produced with a lower quenching rate, 1 K/ps, where atom positions were less relaxed and no shear bands were activated (see Appendix A), the pressure indeed saturates.

Figure 2b shows the trend of hardness as a function of temperature, where the hardness was calculated as an average over contact pressure when it had saturated, i.e., d≥60 Å (see Figure 2a). The pop-ins originally observed in the load-displacement curve caused a momentary decrease in contact pressure because in the moment of slip, the load remains constant while the contact area increases.

In order to evaluate the influence of temperature on the structure we analyzed the von–Mises strain at maximum indentation. Figure 3 shows side views of thin slabs (thickness around 20 Å) centered in the indentation region of the sample at the maximum indentation depth, just before unloading, for temperatures of 0.1 K, 300 K, and 1000 K. The STZs can be identified in these figures as strongly localized regions of increased strain. Such STZs have already been observed in previous simulations [17,38]. We observe shear-band formation on samples at temperatures up to T=300 K. We mostly observe wing-like shear bands, i.e., close to the surface, in particular at T=0.1 K. These wing-like shear bands are marked in Figure 3a. This was particularly noticeable for our lowest temperature (see Figure 3a). We find that the number of STZs strongly increased with temperature; for the largest temperature shown, 1000 K, STZs appeared homogeneously spread over the entire simulation volume at the end of indentation. This was a consequence of enhanced thermally activated relaxation rather than a result of the stress-induced atomic rearrangements as can be confirmed by comparing Figure 3 to figures of the von–Mises strain of unperturbed samples (not shown here).

**Figure 4:**
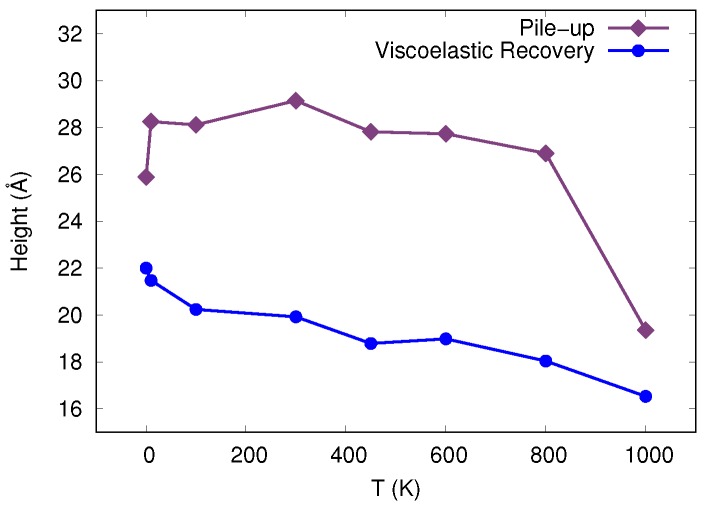
Pile-up height and viscoelastic recovery as a function of temperature. Both quantities are measured after removal of the indenter.

We finally note that with increasing temperature, the strain in the indented region became more homogeneous. Furthermore, at the highest temperature, 1000 K, the pile-up decreased significantly as shown in Figure 4. The pile-up height shown in this figure was determined by the maximum position of the particles in the *z* direction, after the indenter is removed, relative to the height of the simulation box before indentation. The low activity above the surface may be connected to the homogeneous activity of STZs throughout the sample at this elevated temperature.

Additionally, we measure the rebound as a function of temperature after the indenter has been completely removed, also shown in Figure 4. This rebound was determined at the center of the indentation crater as the maximum *z* position of the particles after the indenter is removed relative to the position at maximum indentation depth. The rebound originated from the stress relief during and after indenter removal; thus the compressed material below the indenter can expand elastically. In metallic glasses, in addition viscoelastic strain relaxation did occur [4]; this term included processes occurring on time scales after the indenter was removed and relaxes residual stresses by viscous flow. For simplicity, in Figure 4, we denote the entire rebound as viscoelastic, encompassing elastic and viscous processes. This figure shows the temperature dependence of the recovery. We observed that, for this particular quenching rate, the recovery remained high at all temperatures up to the glass transition temperature; for a discussion of the quenching-rate dependence of this quantity, see Appendix A.

**Figure 5:**
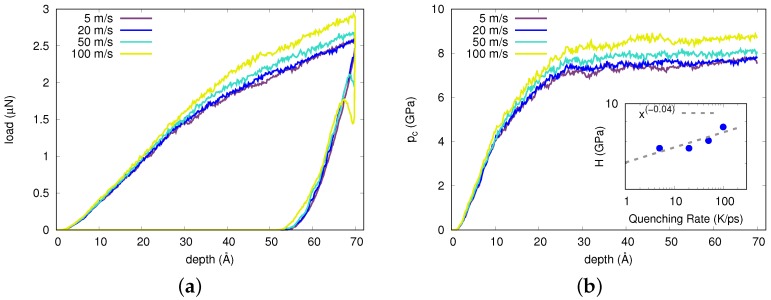
(**a**) Indentation load vs. indentation depth for different indentation rates; all data taken at 10 K. (**b**) Contact pressure as a function of indentation depth for indentation rates shown in (**a**). Inset: Averaged hardness against indentation rate fitted to a power law with exponent ∼0.04.

Furthermore, we studied the effects of the indentation rate on the STZs’ intermittency—i.e., the effects on the serration—during loading and unloading. The load-displacement curve for four different indentation rates between 5 m/s and 100 m/s at 10 K is shown in Figure 5. The figure shows that the load at full indentation increased with increasing rate. Figure 5b shows how the contact pressure evolves as a function of indentation depth for the different indentation rates. The hardness, calculated as the average of contact pressures for indentation depths ≥60 Å, grew as a power law with indentation rate, with exponent ∼0.04 (see inset of Figure 5b). This strain-rate sensitivity was previously reported in nano-indentation experiments [11] and can be explained in the framework of the so-called cooperative-shear model (CSM) [35] where the strain-rate sensitivity is inversely proportional to the activation volume and consequently the STZ volume. Apart from that, the loading curves at all strain rates exhibited different levels of pop-ins which are best observable at higher indentation rates. These results were in contrast to experiments reporting a disappearance of pop-ins at higher strain rates [14,15]. Other experimental studies, however, assigned this effect to a limitation of machinery resolution [18,39].

### 3.2. Analysis of STZs: Non-Affine Squared Displacement

**Figure 6:**
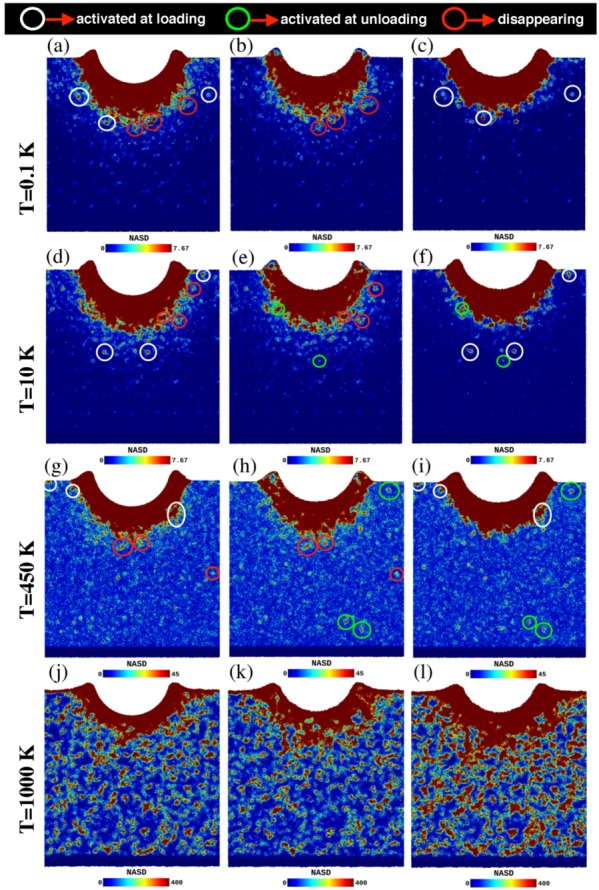
Non-affine squared displacement (NASD)—in units of Å${}^{2}$—at indentation rate of 20 m/s and temperatures of (**a**–**c**) T=0.1 K, (**d**–**f**) T=10 K, (**g**–**i**) T=450 K and (**j**–**l**) T=1000 K. Left column: (**a**,**d**,**g**,**j**) calculated from the atomic configuration at the maximum indentation depth with reference to the atomic configuration before indentation. Middle column: (**b**,**e**,**h**,**k**) calculated from the atomic configuration after unloading with respect to the atomic configuration at the maximum indentation depth. Right column: (**c**,**f**,**i**,**l**) calculated from the atomic configuration after unloading with respect to the atomic configuration before loading. The white circles highlight plastic regions that appear during loading and stay through the unloading process. The red circles enclose plastic regions that were activated during loading, but disappear during unloading, and the green circles feature plastic regions that appear during unloading.

As already mentioned, serration in the force-displacement curve can also be observed in the unloading curve; in this case they are termed pop-outs. In order to study this effect, we calculate the non-affine squared displacement (NASD). The NASD has been introduced in ref. [40] to provide information on the local strain of an atom by comparing its position to that in a homogeneously strained—and hence affine—neighborhood; the NASD hence provides information about non-elastic deformations. Here, we use the NASD—available within OVITO [32]—to identify to what extent plastic rearrangements take place during unloading. For temperatures T=0.1 K, 10 K, 450 K and 1000 K, Figure 6 shows three instances of NASD at different points during the indentation: the snapshots of the first column correspond to the maximum depth of the indentation, just before unloading, where the NASD was calculated with reference to the initial configuration (just before loading), while the other two columns show snapshots after unloading, with reference to the configuration just before unloading (middle column) and to the initial configuration (right column). Two interesting features can be observed in these figures: (i) some plastic regions that were activated during loading seem to be deactivated during unloading, and (ii) new plastic regions appear during the unloading mechanism. In order to help identify these features, we highlight STZs in these figures:

- white circles enclose plastic regions that appear during loading and stay through the unloading process;
- red circles feature zones that were activated during loading, but disappear during unloading;
- green circles emphasize plastic regions that appear during unloading.

The deactivated plastic regions (red) are those that are missing in the left-hand column; this strongly indicates that these regions rearranged to the initial configuration before the indentation. This reversibility has been already observed in amorphous materials [41] and it is even accounted for by the STZ theory [42].

At low temperatures, we could identify only few STZs appearing under unloading (green); for 0.1 K, no region was found in the selected snapshot (see Figure 6b). This agrees nicely with the flat structure of the load-depth curve, Figure 1a, in its unloading part.

At higher temperatures, 450 and 1000 K, we modified the NASD color scale in order to better identify the highly mobile regions. At 450 K, we actually observed many more zones that were activated during unloading than we could highlight in Figure 6h; these activation events correlate with the strong serrations in the load depth curve at high temperature, Figure 1a. In the case of T=1000 K, finally, the density of plastic activity was so high that individual events are hard to detect and therefore, we did not highlight them. However, the strong coloring of Figure 6l—as compared to Figure 6j—demonstrates that also at this temperature, unloading produces many new events, in correspondence with the strong serration observed in the unloading part of Figure 1a.

### 3.3. Analysis of STZs: Cluster Analysis

**Figure 7:**
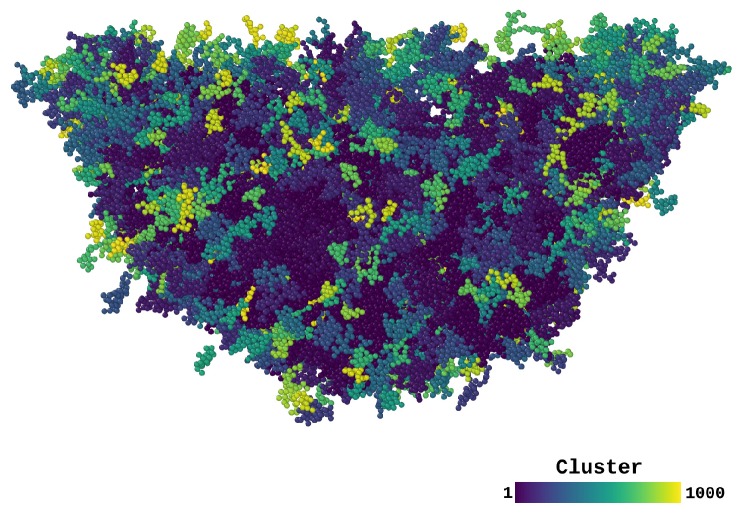
Clusters of atoms with high NASD > NASD${}_{\mathrm{cutoff}}$; these clusters constitute the STZs. Clusters are ordered by size; the largest cluster is dark blue and the smallest cluster is yellow. Only the 1000 largest clusters are shown. Parameters: T=0.1 K, NASD${}_{\mathrm{cutoff}}=20$ Å${}^{2}$ and σ=2.48 Å.

To better study local plasticity during loading and unloading we performed a cluster analysis of the STZs by using their NASD (seen in Figure 6). Similar cluster analysis has been used to study the STZs formed during tensile deformation [43]. Here, we determined their sizes during loading and also measure their evolution during unloading. In order to do this, we first selected the particles with NASD higher than a cutoff value NASD${}_{\mathrm{cutoff}}$. For our analysis we used NASD${}_{\mathrm{cutoff}}=20$ Å${}^{2}$ (see Appendix B for additional discussion). This value corresponds approximately to the square of the second peak position of the radial pair distribution function [27]. In other words, we considered atoms to undergo plastic deformation when its non-affine displacement exceeds the distance of the second nearest neighbors shell. Once the plastically deformed particles are identified, we investigated to which extent they formed clusters; it is known STZs can be characterized as clusters of atoms with high NASD [40]. A cluster-analysis algorithm was available within OVITO [32]. We performed this analysis both at maximum indentation depth and at the final configuration, after unloading. For both cases, we used the atomic configuration before indentation as a reference to calculate the NASD, see Figure 6 first and third column, respectively. We considered a particle to belong to a cluster if it was linked to at least one other particle of the cluster by a separation less than the cutoff distance σ=2.48 Å. This value was less than the one that marks the position of the first maximum of the radial pair distribution $\sigma_{\mathrm{RDF}}=2.77$ Å [27]. Due to the high compression around the indenter, using $\sigma_{\mathrm{RDF}}$ led to a big cluster around the indenter and in turn to the loss of information on the evolution of this region upon unloading, and therefore we chose a smaller value of σ (see Appendix B for additional discussion). Figure 7 shows clusters surrounding the indentation site for T=0.1 K. For clarity proposes, we show in this figure only the biggest 1000 clusters, where the color spectra represents different clusters ordered by size (blue corresponding to the biggest clusters).

**Figure 8:**
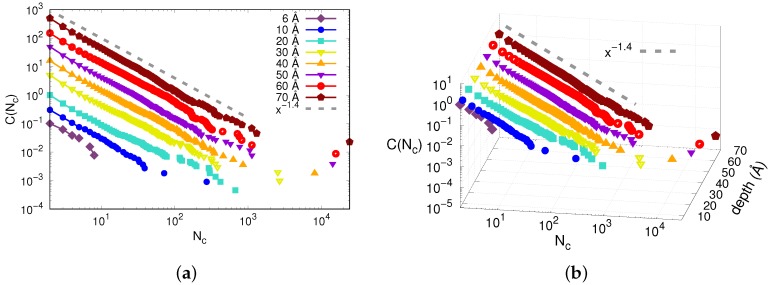
Complementary cumulative distributions $C(N_{c})$ at different indentation depths at T=10 K. (**a**) The curves are shifted in the *y* direction for better readability. (**b**) 3D plot which avoids shifting the curves. The gray dashed line corresponds to a power law with exponent −1.4.

We first present in Figure 8 the complementary cumulative distribution $C(N_{c})$ of the number of particles in the clusters, $N_{c}$, at different indentation depths for temperature T=0.1 K. For the convenience of the reader, we provide available knowledge on $C(N_{c})$ in Appendix C. The complementary cumulative distribution $C(N_{c})$ gives the probability of finding a cluster bigger than $N_{c}$. We can observe that the distributions follow a power law for most of their extension, with exponent ∼−1.4. Such a power law, with an exponent of 1.4, has indeed been predicted by a theory of directed percolation as applied to glass plasticity [24]. The last point of the distribution corresponds to the biggest cluster at the indenter site, which is not of interest here. Also, there is a rapid increase in the maximum cluster size from indentation depth 6 Å to 10 Å, which are both within the elastic regime. The maximum size of the clusters in this regime are consistent with the definition of STZ sizes (∼100 atoms) found in experiments and other simulations [9,10,11]. Beyond the yielding point (∼15 Å for T=0.1 K), the tails of the distribution extend to around 1000 atoms in some clusters, suggesting the presence of shear bands. In the plastic regime, we also observe two different behaviors in the tails of the distributions; a decay for indentation depths 20 Å and 30 Å and an apparent bend toward a smaller power-law exponent for indentation depths ≥40 Å. This shift to a lower power-law exponent has been observed in creep experiments in the distributions of waiting times, where the authors suggest a crossover from STZs to shear banding activity [10,44]. We emphasize that it is our use of large MD samples that allows us to observe such behavior reliably.

We conclude that the size dependence of the distribution followed for all indentation depths a power law with the same exponent. The exponent was the same as the one predicted by statistical mechanics for the temperature dependence of plastic events [24], and was caused by the universality of plasticity in metallic glasses, see Appendix C.

**Figure 9:**
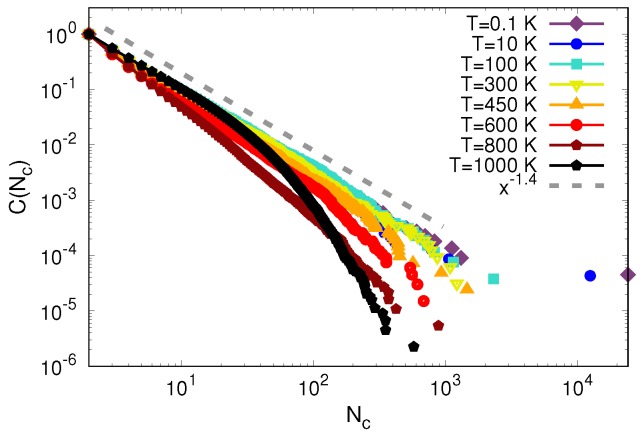
Complementary cumulative distributions $C(N_{c})$ at different temperatures calculated at maximum indentation depth 70 Å. The gray dashed line corresponds to a power law with exponent −1.4.

Furthermore, the complementary cumulative distributions $C(N_{c})$ for different temperatures are shown in Figure 9. Again, we observe a power-law distribution of the cluster size, with an exponent of −1.4, as predicted by the statistical mechanics of glass plasticity [24], see Appendix C. However, deviations from the power-law behavior show up at large cluster sizes, in that the tails of the distributions decay at smaller values of $N_{c}$ when approaching the glass transition temperature. Above the glass transition, T=1000 K, the curve decays very quickly around the value $N_{c}=10$. This points toward independent operations of STZs, i.e., homogeneous flow, which is the expected outcome according to the STZ theory predictions for high temperatures [42]. Overall, our results were consistent with studies suggesting that plastic deformation in metallic glasses occurs via slip avalanches of STZs [10,19,20].

A recent MD study investigated the plastic behavior of a Lennard–Jones glass under uniaxial shear strain [24]. This study identified a transition to percolating clusters with a cluster-size distribution belonging to the universality class of directed percolation [45,46]. Indeed their cluster-size distribution features the same power-law dependence as the one found in the present study. We conclude that the cluster-size distribution found here was quite generic, since it applies both to a simple uniaxial strain loading and the more complex multi-axial loading situation under nanoindentation, and was independent of the interatomic interatomic interaction potentials used (Lennard-Jones or embedded-atom model).

**Figure 10:**
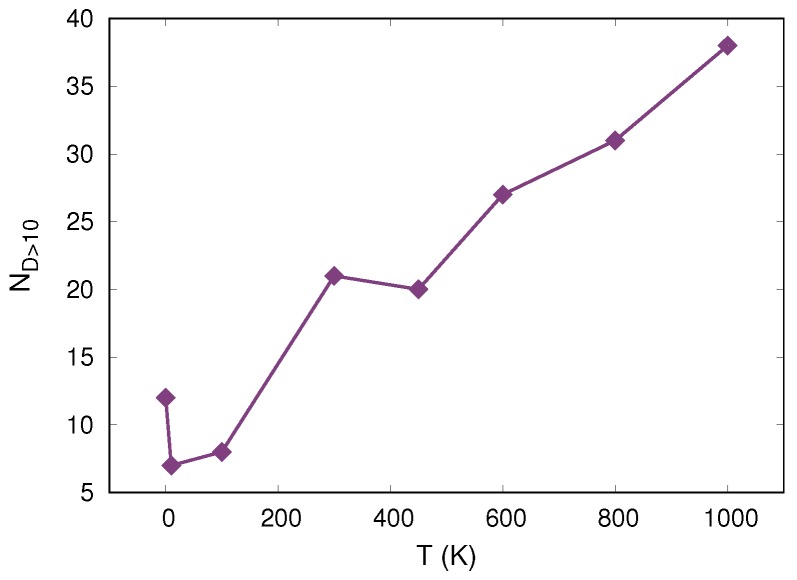
Number of disappearing clusters as a function of temperature.

We then proceeded to make a quantitative analysis on the disappearing clusters and the ones activated during unloading (see Figure 6). We first determined the amount of clusters that disappeared upon unloading for all temperatures. Here, we consider a cluster to have disappeared when it lost more than 90% of its particles. We found these clusters by comparing their configurations at maximum indentation depth (first column of Figure 6) with the configurations after the indenter was removed (third column of Figure 6). For this analysis, we were interested only in clusters that require a large cooperative rearrangement to return to the configuration before loading. Therefore, we considered only clusters with sizes $N_{c}>10$. In Figure 10 we show the number of clusters that disappear, $N_{D}>10$, determined as described above. We found an overall increase on disappearing cluster with increasing temperature and no direct connection with the sample viscoelastic recovery shown in Figure 4.

**Figure 11:**
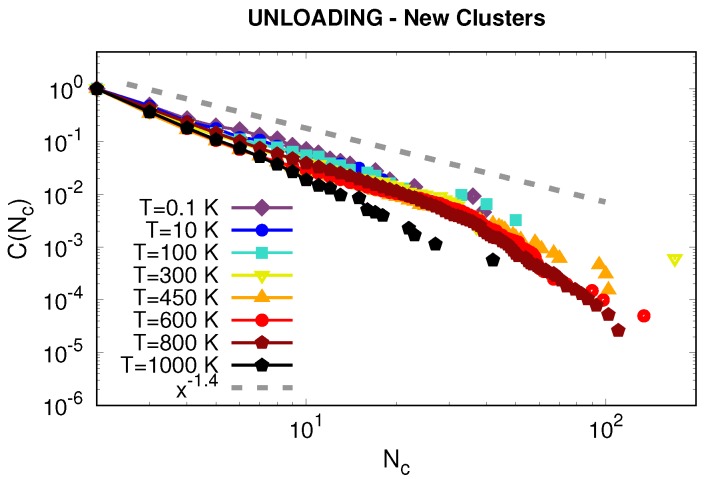
Complementary cumulative distributions $C(N_{c})$ at different temperatures for the activated clusters upon unloading. The gray dashed line corresponds to a power law with exponent −1.4.

Finally, we turned to the study of the STZs appearing upon unloading. We first selected the new particles that have overcame the threshold of NASD${}_{\mathrm{cutoff}}=20$ Å${}^{2}$ upon unloading that did not do so during loading. Then, we determine whether each particle is isolated from already existing clusters (white circles in Figure 6) by using now the criterion $\sigma_{\mathrm{RDF}}=2.77$ Å. We use $\sigma_{\mathrm{RDF}}$ for the unloading analysis since the pressure exerted by the indenter is gone allowing for more space between particles. Finally, we use only the isolated particles and determine cluster formation within them. The complementary cumulative distributions for these clusters activated upon unloading are shown in Figure 11. We can observe in this figure that the extension of the tails is shorter for low temperatures than for high temperatures, meaning smaller STZs at lower temperatures. The sizes of the clusters are ∼100 atoms, which points to STZ operation only during unloading. We also observed that for low temperatures, most of the new clusters are located closer to the indenter site while for high temperatures the regions extend all over the sample (not shown here). Overall, the activity of activated regions during unloading was greater at higher temperatures, which is in agreement with Figure 6 and, as already mentioned, with the strong serration observed in the unloading part of Figure 1a. Moreover, the distributions for all temperatures below the glass transition followed the same power law as the ones calculated at maximum indentation depth. This finding strongly suggests that the STZs formed upon unloading operate with a similar mechanism as upon loading. To our knowledge, STZ formation during unloading has not been reported before and it is important to study the impact of newly forming STZs on the global deformation mechanism.

## 4. Summary

We summarize the main findings of our atomistic study on plastic activity during nanoindentation of a CuZr glass as follows.

1. The yield point, elastic modulus and viscoelastic rebound of the metallic glass decrease with increasing temperature. This behavior is in qualitative agreement with predictions of the cooperative shear model [35,47].
2. Material hardness weakly increases with strain rate, confirming previously reported experimental findings and the predictions of the cooperative shear model [11,35].
3. Glass plasticity leads to a serrated structure of the load-displacement curves, in agreement with experiment [14,15]. For the loading curve, we do not observe a strong dependence on temperature.
4. An analysis of the plastic activity using the technique of non-affine displacements [40], together with a cluster analysis, allows us to identify the STZs created during the indentation process [48]. The complementary cumulative distribution of plastically deformed regions, $C(N_{c})$, follows a power law with an exponent around −1.4. This power-law of $C(N_{c})$ found here is consistent with that found previously in uniaxially sheared Lennard-Jones glass and points at a percolation transition in the formation of STZs [24].
5. Surprisingly, the power exponent which has been found theoretically to describe the temperature dependence of plasticity, also describes its depth dependence. In addition, the power law found during loading is the same as for the new clusters that form during unloading.
6. We observe an apparent transition from STZ operation to shear banding. This shear band activity is observed at room temperature and below, and is particularly strong a the lowest temperature simulated, 0.1 K.
7. During the unloading process, part of the created STZs deactivate and also new zones are created. The number of large disappearing STZs increases with temperature. The amount of plastic activity and STZ sizes during the unloading process strongly increases with temperature, and correlates well with the increased serration of the unloading curve at increased temperatures. In this context, the pop-outs formed in the unloading process have the same nature as the pop-ins found during loading.

## Figures and Tables

**Figure A1:**
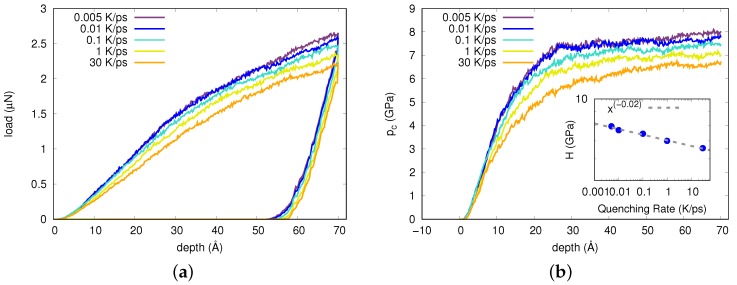

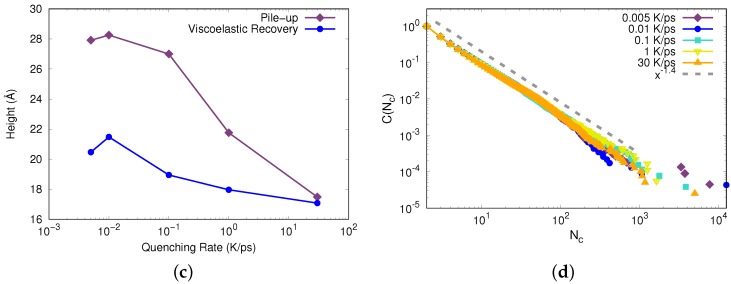
Quenching-rate dependence in nanoindentation; all data taken at 10 K. (**a**) Indentation load vs. indentation depth for various quenching rates. (**b**) Contact pressure as a function of indentation depth for the quenching rates shown in (**a**). Inset: Averaged hardness against indentation rate fitted to a power law with exponent ∼-0.02. (**c**) Pile-up height and viscoelastic recovery vs. quenching rate. Both quantities are measured after removal of the indenter. (**d**) Complementary cumulative distributions $C(N_{c})$ for the quenching rates shown in (**a**). The distributions were calculated at maximum indentation depth 70 Å. The gray dashed line corresponds to a power law with exponent -1.4.

**Figure A2:**
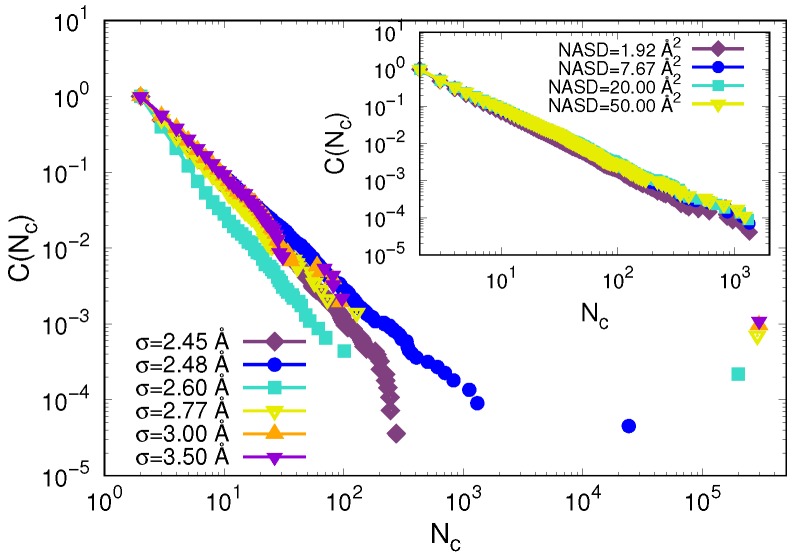
Cumulative distribution $C(N_{c})$ of cluster sizes $N_{c}$ for different values of σ. Inset: Cumulative distribution $C(N_{c})$ of cluster sizes $N_{c}$ for different selections of the NASD parameter NASD${}_{\mathrm{cutoff}}$.

## Appendix A. Quenching-Rate Dependence

It is known that the quenching rate used in sample preparation plays an important role in the way metallic glasses deforms under loading [26,28]. Lower quenching rates leads to more localized plasticity and are therefore desirable for the study of shear-band formation [26]. In this section we study the dependence on quenching rate for some of the quantities presented in the main text. In this section, we fix the temperature at T=10 K and the indentation rate at 20 m/s, while the quenching-rate varies and is chosen as 0.005 K/ps, 0.01 K/ps, 0.1 K/ps, 1 K/ps and 30 K/ps.

In Figure A1a we show the load–indentation-depth curves. We observe that the behavior in the elastic regime is very similar for all quenching rates; in the plastic regime, the load increases more rapidly for lower quenching rates. The contact hardness (shown in Figure A1b) increases with lowering the quenching rate. The averaged values of the contact hardness, shown in the inset of Figure A1b), were calculated after indentation depth 60 Å and fitted to a power law with exponent ∼−0.02.

The dependence of the height of the pile-up and the viscoelastic recovery for the different quenching rates is presented in Figure A1c. Both quantities show the same trend: they decrease when the quench rate increases.

Moreover, we perform the cluster analysis introduced in the main text to calculate the STZ sizes during loading for the different quenching rates. The complementary cumulative distributions shown in Figure A1d follow the same power law up to $N_{c}\approx 100$; above this cluster size, the tails of the distributions change showing bigger plastic events for lower quenching rates. The changes in the distributions are not so strong for different quenching rate as for different temperatures (see Figure 9). Therefore, we conclude that a choice of an even lower quenching rate as the one adopted in this study, 0.01 K/ps, would not significantly affect the main results of our manuscript.

## Appendix B. Cluster-Analysis Parameters

In this section we present our reasoning for the selection of the parameters σ and NASD${}_{\mathrm{cutoff}}$ used for our cluster analysis in this manuscript.

We first present how the complementary cumulative distributions change for different values of NASD${}_{\mathrm{cutoff}}$. In the main text we explained that this parameter determines the threshold for plastic activity. So, we consider any particle with NASD above this threshold to have undergone plastic deformation. The inset of Figure A2 shows minimal changes in the distributions in the NASD${}_{\mathrm{cutoff}}$ range from 1.92 Å${}^{2}$ to 50 Å${}^{2}$.

Furthermore, we examine the effects of different selections of σ values. Figure A2 presents the cumulative distributions for the number of particles in the clusters, $N_{c}$, for different values of σ. One can observe that the tails of the distributions extend to higher cluster sizes from σ=2.45 Å to σ=2.48 Å which may be expected since it is more likely to find more particles for a larger value of σ. However, for even higher values of sigma the distribution ends at smaller values of $N_{c}$, except for one point. This point represents the indenter site which has became a huge cluster and therefore we lost information about cluster formation around the indenter. We therefore select σ=2.48 Å for the present study, as it allows us to obtain information about both, the indenter site and its vicinity.

## Appendix C. Statistics of STZs

In this section, we review the statistical mechanics of STZs as studied via the concept of avalanche dynamics applied to the plastic deformation of metallic glasses [20]. For a more specialized discussion on the subject, we refer the reader to [49]. In general, the distribution of cluster sizes $N_{c}$ follows the law

(A1) $$D\left(N_{c}\right)=N_{c}^{-\tau }f(N_{c}|T-T_{c}{|}^{1/\sigma }),$$

where σ and τ are critical exponents, $T_{c}$ is the critical temperature and the universal scaling function f(x) is expected to decay exponentially as f(x)=Aexp(−Bx), with *A* and *B* being non-universal constants. Away from the critical temperature $T_{c}$, the distribution follows the same power law up to a maximum cluster size

(A2) $$N_{c}^{max}∼{\left|T-T_{c}\right|}^{1/\sigma }.$$

This means that the further away from the critical point $T_{c}$, the faster the decay of the distribution.

In this manuscript, we study the complementary cumulative distribution function, which is related to the distribution of cluster sizes, Equation (A1), as

(A3) $$C\left(N_{c}\right)=\int_{N_{c}}^{\infty }D\left(N_{c}^{\prime }\right)\cdot d\;N_{c}^{\prime },$$

which gives the probability to observe clusters larger than size $N_{c}$. For small $N_{c}$, the cumulative distribution $C(N_{c})$ decays as

(A4) $$C\left(N_{c}\right)∼N_{c}^{-(\tau -1)}.$$

The critical exponent assumes the value τ=2.4 according to [24].

Therefore, we expect the complementary cumulative distribution of active regions to follow a power law with the power −(τ−1)=−1.4 for small values of $N_{c}$; for larger $N_{c}$, the distribution will be cut off at a value which depends on temperature, Equation (A2).

In the case of indentation depth, we replace $|T-T_{c}|$ for $|d-d_{c}|$ in the above equations.
